# Appraisal of the national response to the caries epidemic in children in Nigeria

**DOI:** 10.1186/1472-6831-14-76

**Published:** 2014-06-23

**Authors:** Morenike O Folayan, Nneka M Chukwumah, Nneka Onyejaka, Abiola A Adeniyi, Olubukola O Olatosi

**Affiliations:** 1 Department of Child Dental Health, Obafemi Awolowo University, Ile-Ife, Nigeria; 2 Department of Child Dental Health, Obafemi Awolowo University Teaching Hospitals Complex, Ile-Ife, Nigeria; 3 Department of Preventive Dentistry, Lagos State University College of Medicine, Ikeja, Lagos State, Nigeria; 4 Department of Child Dental Health, College of Medicine, University of Lagos, Idi-Araba, Lagos, Nigeria

**Keywords:** Caries, Epidemic, Global response, Nigeria, Children

## Abstract

**Background:**

This article reviews the caries profile for children in Nigeria and proposes an appropriate framework for addressing the silent caries epidemic.

**Discussion:**

We reviewed the caries prevalence among children in Nigeria, assessed the existing responses to the caries epidemic including the national oral healthcare delivery situation in the country and discussed the current caries management in children. We then proposed a response framework for Nigeria. We argue that successful interventions will require the adoption of a socio-ecological model. This would ensure that the micro-, meso-, exo- and macrosystems required to support the behavioural, structural and biological interventions for promoting caries prevention are addressed. National oral health surveys are required to help understand the epidemiology, social determinants of and factors that undermine the ability of children to access oral health care. A global caries prevention agenda for children would help get the government’s support for a national response agenda. Currently, there is no global call for action on the caries epidemic in children. This lack of an agenda needs to be urgently addressed.

**Summary:**

A combination of approaches for the prevention of caries in children in Nigeria is needed. A national survey is needed to generate the needed evidence for the planning of community relevant responses to the national caries epidemic in children. The design of a global health agenda for children is an important first step that can facilitate the development of a national oral health programme for children in Nigeria.

## Background

Research on dental caries in Nigerian children has been conducted for several decades. The prevalence of caries in the early 1980s was high [1-4]. However, the prevalence of dental caries varies with the study location in Nigeria, ranging between 13.9% to 17.4% in the semi-urban settlement of Ile-Ife [5-7] to between 11.2% and 48.0% in urban areas such as Benin [4,8,9], Enugu [10-12], Lagos [1,2,13-21] and Ibadan [3,22,23]. The prevalence of caries is higher in urban than in rural areas [24-27], higher in Northern than in Southern Nigeria [25] and higher in primary than in permanent dentition [6,22,25]. This prevalence is of epidemic proportion because it is higher than the 5% epidemic threshold. See Table 1. Unfortunately, a trend analysis of the prevalence of caries over time has not been possible because studies were conducted in different age groups, using different methods, and in diverse populations. The only national data on the prevalence of dental caries in children in Nigeria was conducted in 1995, and showed prevalence as high as 30% and 43% in children aged 12 years and 15 years respectively [27]. However, the evolving evidence points to the need for proactive action to address the dental caries epidemic among children in Nigeria, especially for caries affecting the primary dentition. A previous study conducted in Lagos, Nigeria showed that despite intervention, the prevalence of caries in primary dentition increased from 17.9% to 20.4% over a 3-year period, though there was a 34.8% decline in the prevalence of caries in permanent dentition over the same period [21].

**Table 1:** Prevalence of caries in population-based surveys in Nigeria

**Nos.**	**Authors**	**Study location**	**Characteristic of study location**	**Year of publication**	**Age group studied**	**Caries prevalence**
1	Akpata [1]	Lagos	Urban	1979	6 yrs	40.0%
2	Adenubi [2]	Lagos	Urban	1980	4 yrs and 5 yrs	
3	Idiakhoa [13]	Lagos	Urban	2001	5 yrs and 12 yrs	22.5%, 15.8%
4	Sho-Silva [14]	Lagos	Urban	2004	3 yrs – 10 yrs	24.0%
5	Giwa [15]	Lagos	Urban	2005	12 yrs	5.7%
6	Agbelusi and Jeboda [16]	Lagos	Urban	2006	12 yrs	24.6%
7	Umesi-Koleosho et al [17].	Lagos	Urban	2007	11 yrs - 16 yrs	23.8%
8	Sowole & Sote [18]	Lagos	Rural and urban	2007	6 months - 5 yrs	10.5%
9	Abiola AA et al [19]	Lagos	Rural and urban	2009	18 months – 5 yrs	10.9%
10	Adeniyi et al [20]	Lagos	Urban	2012	5 yrs - 16 yrs	13.1%
11a	Sofola et al [21]	Lagos	Urban	2014	2 yrs -12 yrs	2000 -17.9%
11b	Sofola et al [21]	Lagos	Urban	2014	2 yrs -12 yrs	2003 - 20.4%
12	Alakija [4]	Benin	Urban	1983	5 yrs – 14 yrs	40%
13	Okeigbemen [8]	Benin	Urban	2004	12 yrs - 15 yrs	33.5%
14	Chukwumah et al [9]	Benin	Urban	2012	7 yrs – 15 yrs	15.7%
15	Noah [3]	Ibadan	Urban (private schools)	1981	6 yrs	46.0%
16	Noah [23]	Ibadan	Urban (public schools)	1984	6 yrs	18.0%
17	Denloye [22]	Ibadan	Urban	2005	12 yrs - 14 yrs	11.2%
18	Ojofeitimi et al [28]	Ile-Ife	Semi-urban	1984	8 yrs – 15 yrs	32.2%
19a	Kubota et al [7]	Ile-Ife	Rural and semi-urban	1990	**1981:**	
					Urban:6 yrs-8 yrs	33.6%
					Rural:6 yrs-8 yrs	30.5%
					Urban:9 yrs-11 yrs	33.4%
					Rural:9 yrs-11 yrs	28.8%
					Urban:12 yrs-14 yrs	28.1%
					Rural:12 yrs-14 yrs	34.6%
19b	Kubota et al [7]	Ile-Ife	Rural and semi-urban	1990	**1986:**	
					Urban:6 yrs-8 yrs	42.7%
					Rural:6 yrs-8 yrs	5.8%
					Urban:9 yrs-11 yrs	38.4%
					Rural:9 yrs-11 yrs	14.8%
					Urban:12 yrs-14 yrs	29.8%
					Rural:12 yrs-14 yrs	9.2%
20	Adekoya - Sofowora et al [5]	Ile-Ife	Semi-urban	2006	12 yrs	13.9%
21	Ozeigbe & Esan [6]	Ile-Ife	Semi-Urban	2013	4 yrs - 6 yrs	17.4%
					7 yrs – 12 yrs	10.1%
					13 yrs -16 yrs	7.5%
22	Udoye et al [10]	Enugu	Urban	2009	12 yrs - 15 yrs	24.1%
23	Okoye et al [11]	Enugu	Urban	2010	12 yrs - 15 yrs	15.5%
24	Okoye& Ekwueme [12]	Enugu	Rural	2011	11 yrs - 16 yrs	35.5%

The severity of caries is low in Nigeria. In the permanent dentition, the decayed-missing-filled teeth (DMFT) ranges between 0.02 and 0.85 [5,6,8,10-21]. While the severity of caries is also low in primary dentition, a dmft index of greater than 1.0 is often only recorded in the primary dentition [3,13,21] in children from urban areas. An exception to this finding was found in the study by Ojofeitimi et al. [28] who reported a dmft greater than 1.0 in children who resided in a semi-urban region of Nigeria.

Of major concern is the high level of untreated caries in the permanent dentition. The proportion of children with untreated caries ranges from 77.2% in Ile-Ife [5], to 98.6% in Benin [8] and 49.5% to 85.5% in Enugu [10-12]. In Lagos, the restorative index is 1% [17] and the Met Need index in Ibadan is 0.11 [22]. The prevalence of untreated caries in the primary dentition is also high, with values above 80% in all parts of Nigeria [6,29].

Despite the high need for dental treatment, dental service use continues to remain low and is often prompted by oral symptoms such as pain, and the need for curative treatment [30-36]. Perception about the need for dental service use for preventive oral healthcare and management of oral health problems that are not associated with pain is also low [34]. However, there is little evidence-based information on how dental service use for preventive oral healthcare and prompt management of oral caries can be increased. Ola et al. [36] showed that pupils whose parents had a university degree were 70.0% more likely to visit the dentist in the last year than those whose parents had no university degree. Pupils that attend non-fee paying schools are three times more likely to visit the dentist for preventive reasons than those that attend high fee paying schools. Additionally, pupils living with one or both parents visit the dentist more often for preventive reasons than those living with persons other than their parents. School programmes could also improve use of oral health care services for curative purposes [34]. Referral from the paediatric clinic did not increase dental service uptake [37].

The use of recommended oral self-care (twice-daily tooth brushing, use of fluoridated toothpaste and avoidance of consumption of refined carbohydrates between meals) for the prevention of caries is low with only 7.8% of children from Southern Nigeria practicing recommended oral self-care [35]. A large number of children consume sugar in between meals and more than once a day, do not brush twice-daily and do not use dental floss [35]. These statistics highlights the magnitude of the problem with caries prevention for children in Nigeria.

The vision of the revised national oral health policy [38] launched in 2012 is: *To promote optimal oral and general health for all Nigerians, reduce the morbidity and mortality rate, as well as reverse the increasing prevalence and incidence of oral diseases; to meet the global targets on the elimination and eradication of oral diseases and significantly ensure the maintenance of complete set of dentition through life, thus promoting general health for all Nigerians.*

To achieve this vision over time, a review of the current structure and systems for providing oral healthcare service for children in Nigeria is important. Therefore, we investigated models for providing oral healthcare services for children that would reduce the current prevalence of caries and promote the use of dental services for preventive care and prompt disease management.

To address the study objectives, we reviewed articles published in peer-reviewed journals, documents from international agencies, such as the World Health Organization, Internet resources and research uploaded in the Nigerian scientific database that provided insight into the epidemiology of caries among children in Nigeria and addressed the objectives of the study. Studies that were included were those that reported on the prevalence and severity of dental caries, and those that reported on dental service use. To ensure the validity and reliability of the information obtained, we examined the information for consistency, and whenever possible, verified it by triangulating it with data in other documents. Information that could not be fully substantiated was excluded.

The first strategy was a search of PubMed, Global Health and African Journal online databases for relevant information. The initial search resulted in over 12,000 references being identified using the search terms “caries”, “children”, “epidemic”, “prevalence”, “response” and “Nigeria”. A review of these articles showed some duplication, as well as inclusion of materials that were not relevant to the study. All of the data on the prevalence of caries for this study were limited to population-based studies. All hospital-based data were excluded from the analysis on caries prevalence. Only 49 of these articles were deemed relevant to the study objectives. One study was excluded from the analysis because we determined that the methodology was faulty [39]. A second study was excluded because of inconsistencies in the data presented on the prevalence of caries for the study population [40]. A third study was excluded because the data were derived from a secondary analysis of a prior data reported [41]. A fourth study was excluded because it was a repeat publication [42]. A fifth study was excluded because the study was not conducted methodologically and a detailed oral examination was not performed [43]. Three further studies were excluded because efforts to retrieve them were unsuccessful [44-46]. The prevalence of caries reported in the study by Kubota et al. [7] was recalculated to be able to obtain the population level prevalence of caries. This also allowed for data comparison and analysis.

Searches were then performed on the websites of organisations, such as the Nigerian Federal Ministry of Health ( http://www.fmh.gov.ng), the WHO ( http://www.who.int/) and the World Federation of Dentists ( http://www.fdiworldental.org). We identified an additional 13 materials for inclusion in the appraisal. We then checked the reference lists of all documents and articles retrieved in the previous search strategies to identify relevant materials. This retrieved a further 36 papers that were not previously included. We used the generic search engine Scirus to source additional information as necessary.

Finally, the database of the National and West Africa Postgraduate Medical College Fellowship examination thesis on caries, caries prevention and caries management in children was reviewed.

To study service delivery models, search words included “health service models”. Where appropriate, the “related articles” search tool was used to retrieve more relevant materials.

## Discussion

### Significance of dental caries in children

Dental caries has major implications for the overall health and well-being of the child. This is because of the intimate relationship between oral health and general health, and the association between caries and mortality. When caries is left untreated, it can lead to infection [6], which can spread to other parts of the body, including the brain [47]. There have been two reported cases of children dying from brain abscess resulting from untreated caries [48,49]. Many more children, especially in a developing nation such as Nigeria, might have died from dental problems since reports are often inaccurate because caries directly leads to infection, but is not ultimately defined as the cause of death [50]. Caries is also associated with multiple morbidities, and with social, psychological, health and economic consequences. For children, the persistent pain from untreated caries decreases the quality of life of the child, interrupting the ability to learn, play, eat and sleep. As shown by Elice et al., severe caries can contribute to a child failing to thrive [51]: children with caries weigh significantly less than their peers [52].

Although children who suffer from chronic pain may live fulfilling and productive lives, the pain makes it more difficult for them to do so [53]. Persistent pain causes the child to lose school hours, and when in school, the time spent is markedly less productive [53]. This is because pain interferes with the ability of the child to concentrate. Pain is exhausting and diminishes the ability of the child to take on the task of a full day of school. Pain also reduces the ability to eat healthy food [54]. Unfortunately, poor feeding exacerbates the effects of pain on concentration and energy, and leads to malnutrition [55]. The effect of pain from caries on the ability of the child to play and participate in other childhood activities may also disrupt the development of the child’s psychological capacity because this affects intellectual development and other social skills provided by the activity [56-58].

### Appropriate framework to design interventions for elimination of diseases

Caries is a preventable disease. Brushing with fluoride containing toothpaste daily, dental flossing, seeing a dentist at least once a year for disease prevention and prompt detection of early lesions, reduction of sugar consumption and elimination of in-between meal snacks are important and effective caries risk-reduction strategies that require behaviour adherence. Any approach developed for addressing oral healthcare issues in children should therefore be based on a social ecological model.

Social ecological perspectives posit that individual behaviours are influenced by individual characteristics, interpersonal processes and macro-level contextual factors. The model is a framework that recognises the multiple effects and interrelatedness of social elements in an environment. It allows for the integration of multiple level contexts to establish the overall health picture [59]. Such interventions would ensure that micro-, meso-, exo- and macrosystems are all addressed simultaneously in a bid to promote the preventive oral healthcare needs of the child.

At the microsystems level, interventions should enhance the process of learning oral health behaviours. However, the learning process should be sensitive to issues related to personality, knowledge and beliefs of the child. Attention also needs to be paid to significant others like family, groups of friends and other social contacts who have multiple simultaneous influences on children’s behaviour and learning.

At the mesosystems level, interventions should focus on norm-forming organisational or institutional factors such as rules and policies that shape or structure the environment within which the individual lives and interpersonal relations occur [60]. Examples of mesosystems include schools, churches, mosques and sports teams. This component is especially influential with young children. Bronfenbrenner noted that the richer the medium for communication in this system, the more influential it is on the microsystem [61].

Exosystems refer to the influence of the community, including their fairly established norms and standards [60]. The community is a web of many organisations and interpersonal relationships, larger than the mesosystem yet considerably smaller than the respective nation or culture it comprises. Exosystems are essentially any setting, which affects the individual, although the individual is not required to be an active participant [60]. Interventions at this level would seek to change norms and standards that influence oral health practices negatively and reinforce those that influence oral health practices positively.

**Figure 1:**
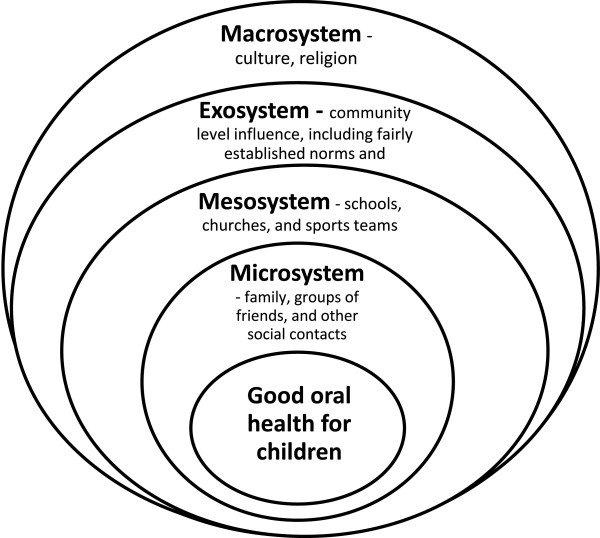
Diagrammatic representation of the appropriate framework for designing interventions for the elimination of disease.

Macrosystems are the cultural contexts in which an individual exists. They are geographical or physical, as well as emotional and ideological [60]. These influences are more easily seen than the other factors, mainly owing to the magnitude of the effect. Examples of considerable intercultural effects include Western culture, Islam and Christianity. Figure 1 gives a schema representation of this framework.

Understanding this framework and using it to inform the design of an intervention could ensure success of planned intervention. This is because a child’s health-related attitude and behaviour are taught and adopted at home, and modelled on parental and family examples through the primary socialisation process [62]. Later in life, these attitudes and behaviours are influenced and shaped by mesosystem level factors and are formalised in a community-based network (the exosystem). This process is called secondary socialisation [63]. When a child’s adopted norms through the primary socialisation process differs significantly from those adopted in a school, he or she faces difficulty with adopting the new behaviours [64]. Therefore, systems need to be in place to support children adopting new caries preventive behaviours. The subsequent section discusses how supportive systems and structures can be put in place to promote the adoption of preventive behaviour in children, including those that take into account the context specific issues in Nigeria.

### Appropriate interventions for the Nigerian child

#### Interventions for modifying behaviour

There is a long- history of efforts to modify behaviour to ensure a reduction in the risk of caries. These behaviour modification efforts helps children learn to practice oral hygiene and maintain good oral health, limit sugar intake and encourage regular visits to the dental clinic. However, modifying behaviour is a difficult and slow route for change [65,66]. Therefore early intervention prior to formation of behaviour is a more appropriate approach especially when working with children.

Several studies have shown that behaviours are adopted at a young age through daily interactions with parent(s) or caregiver(s) [67,68]. These formed behaviours are likely to be sustained to the teenage years [69-73]. In late adolescence, parents and peers have influential roles on behaviour. Parents’ disapproval of risky behaviour contributes to their children’s healthy lifestyles, while peer approval of risky behaviour contributes to unhealthy lifestyles [74]. Therefore, reaching out to parents is critical for the success of any oral health behaviour modification efforts for children. Parental health is also critical for successful individual interventions because aversive parental experiences and disregard for primary dentition are serious obstacles for preventing early childhood caries [75].

Studies in Nigeria had established associations between poor oral hygiene [76], the frequency of sugar intake [5], maternal attitude and the risk for caries [75]. Oral hygiene practices and sugar intake by children are often shaped by maternal practices [77]. It is therefore important to effectively engage parents and or caregivers to be able to promote the development of healthy oral health habits early in the life of the child. Interventions directed at parents and caregivers should provide education on the “how” and “why” of developing good oral health habits in children. Interventions that can enable caregivers support their children to adopt health oral health behaviour early in life are discussed later.

School based behaviour modification programmes introduce children to oral health care at a time when habits are formed. Where school programmes do not actively engage parents, the home reinforcement needed to bring about a change in behaviour may be lost. Unfortunately, school programmes in Nigeria are limited to providing information on oral health care to the children, conduct of oral examinations and referral for disease management. This approach results in slight increase in use of oral health services for mainly curative purposes [21,34].

School based oral health programmes have been shown to be successful in reducing caries incidence [78]. These programmes however do much more than education; they are sustained interventions that provide oral health services within the school at very low cost where it is not possible to provide free treatment. This way, oral health care is provided in close proximity to the child eliminating the (1) potentials for children to be lost from care when referred for services [79], (2) challenges associated with health access due to distance [80] and (3) cost of travel [81]. It is important to pilot this model of school based oral health programmes in Nigeria so as to document its effectiveness and impact on oral health behaviour modification and dental service uptake for both curative and preventive purposes.

School based oral health programmes in Nigeria would only reach 60.0% of primary school aged children and 40.0% of secondary school aged children [82]. Complementary efforts to reach children needs to be sought. For this reason, prime importance needs to be placed on supporting parents/caregivers who have challenges with providing support for their children because of personal health, financial or other reasons through structural interventions.

#### Structural interventions for modifying the environment

Structural interventions would require that children’s caregivers are empowered with information about caries management. One of these interventions is to design perinatal educational programmes, which provide information on maternal oral health and perinatal care for the child. Mother’s oral health throughout pregnancy helps establish a foundation to promote good oral health for the mother and child after birth [83]. Structures already exists that address maternal education in hospitals during antenatal visits. Oral health education and prevention programmes for the mother and child can easily be incorporated into these structures.

Perinatal maternal oral health education programmes should however, not be limited to the hospitals as only 64% of pregnant women receive antenatal care services [84]. In some regions of Nigeria, up to 67% of pregnant women do not receive antenatal care services [84]. Considerations should be given to implementing programmes with traditional birth attendants and mission homes where a large number of pregnant women in Nigeria receive perinatal care [85].

For the long term sustainability of perinatal education of pregnant women on oral health, it is important to explore the inclusion of oral health programmes into maternal and child care programmes in Nigeria using the common risk factor approach [86]. Maternal and child care programmes in Nigeria have received a lot of support from national and international agencies because of the poor national maternal and child health indices [87]. This would require stakeholders involved with oral health care to consider the possible role of a common risk approach for oral and systemic disease prevention for maternal and child health in Nigeria. This approach would promote multidisciplinary meetings and inter-professional collaborations.

Furthermore, oral health is intimately linked to national policies. Policies that address the political and economic processes that cause poverty and disparities in oral health may reduce health risks and financial barriers to dental care [88]. It should also support improved access of children to education as well as restrict sugar access. Sugar restrictive policies should promote use of sugar substitutes for medication by pharmaceutical and confectionery companies and reduce access of children to cariogenic snacks in-between meals at school by banning sale and access to cariogenic beverages and meals in schools [89].

The implementation of this policy requires the establishment of a complimentary public health programme that facilitates public education and access to preventive oral health measures. One of such programmes to be considered is the deployment of trained dental therapists to underserved areas which might help address the challenges with personnel and reduce the current inequities in child access to oral healthcare. Dental therapists can play this role since they are trained to provide comprehensive primary care to school children. Another programme to explore is for community health extension workers (CHEW), who are trained healthcare professionals already working closely with local residents, to integrate oral health education into ongoing health education programmes. However, revision of the current education curriculum of CHEW in Nigeria to include details on oral health education would be required.

Programming for rural oral health care could lead to community-organised efforts to address forces that contribute to unequal oral health access and subsequently induce change at the individual level [90]. Coordinated community efforts with already existing school and community programmes, such as Parent Teacher Associations and local community centres activities, can enhance such efforts. One such existing community health programme structure is the Ward Development Committees (WDC) established by the Nigerian Government to provide support for health delivery services in all political wards in Nigeria: they mobilise local community resources, promote health and increase demand for health services necessary for enhancing access by families, households and the communities. The WDC represents the closest administrative link between health facility services and the communities. Through this structure, community gatekeepers are directly involved in the management of community-based services in ways that contribute to sustainability of the primary health care interventions. The WDC has been effectively engaged for the provision of HIV/AIDS, Tuberculosis and malaria services in Nigeria [91]. The potential for this structure to promote oral health care in general and oral health care for children specifically needs to be explored.

In the absence of structural interventions, disparities in child access to health will continue. Disparity arises from a web of effects, which include complex cultural, economic, social, biological, behavioural and political processes that affect oral health and access to effective oral healthcare. For children, evidence has increasingly shown that their health is affected by the socioeconomic circumstances in which they grow up. Higher rates of debilitating disease have been found in poorer communities. Poor children suffer nearly 12 times more restricted-activity days than those from higher-income families, and 8% of children aged 2–5 years bear the burden of 75% of caries reported for their age group [92,93]. Unfortunately, childhood health disadvantages compromise health in adult life [94]. Childhood disadvantage has been linked to an elevated risk of early death from coronary heart disease, stroke and respiratory disease [95].

For structural changes to be made, understanding what these disparities are, what causes them, and how to ameliorate and prevent them are important. This will require increasing the current level of awareness, research to generate evidences, and the translation of these evidences to action. This also requires the willingness to act by all of those engaged with the provision of oral health services for children for change to occur. Changes in the social and public health policy, community organisation, provision of effective dental healthcare, and change in professional and individual behaviour are required to make any considerable impact in oral care provision for children. Changes also have to occur in the way resources are allocated to oral health care. Currently, the Nigerian government spends about 70% of its budget in urban areas where 30% of the population resides: an investment profile that is inversely related to the need of the population [96]. Of this, only about 0.41% of the national health budget is allocated to oral health [97]. Worse still, very few primary health care centres – the closest unit of health care service provision - provide oral health care services [97]. Where oral health care services are provided, the quality of care is poor resulting in large proportion of the population suffering neglect [97].

#### Biological intervention for modifying the host (or the pathogen)

There are multiple examples of biological interventions that have helped modify the host and change the epidemiological profile of diseases in history. For dentistry, fluoride is a biological intervention that has successfully modified the host response to cariogenic pathogens. Topical application of fluoride has resulted in a significant reduction in the incidence of caries [98]. Fluoride has been shown to be highly efficacious in reducing caries because of multiplicity of topical and systemic actions [99-102].

The impact of fluoride on the epidemiology of caries in Nigeria has not been documented. The fluoride content of water in various parts of the country ranges from 0.3 ppm to as high as 6.7 ppm [103] with wide variations noted within the same geographical zones. Only 21% of local government studied had the appropriate recommended level of fluoride in drinking water for tropical countries [103]. Water fluoridation programme in Nigeria will have very limited success as less than 10% of the Nigerian population have access to tap water [103].

One single effort to improve access of Nigerian children to fluoride is the effort to ensure the use of fluoridated toothpaste. There is a high likelihood that the widespread use of fluoridated toothpaste may have been responsible for the decline in caries prevalence in children recorded in the 80s’though there is no evidence to support this hypothesis. About 83% of children use fluoridated toothpaste in Southern Nigeria and its use is commoner with younger children than older children [35] pointing to a tendency to increasing interest and public support for use of fluoridated toothpaste. The increased public access to fluoridated toothpaste has been supported by the National Agency for Food Administration and Control policy wherein national approvals for marketing and sales of toothpastes are only given to those toothpastes that contain fluoride.

The use of fluoride toothpaste still needs to be complemented by other efforts. One of this is the promotion of widespread use of fissure sealants. Over 80% of the caries lesions in children are occlusal caries [25]. Fissure sealants are more effective in the prevention of prevention of occlusal caries than topical fluoride made available through dental dentifrices or water fluoridation programmes. There is high support for the use of pits and fissure sealants as a caries prevention tool in Nigeria [104]. Access to the product and skills to use fissure sealants may be limitations to its widespread use.

### Efforts needed to facilitate the caries control programme for children in Nigeria

There clearly is a caries epidemic in Nigeria just like there is in many other countries around the world [58]. Well over 5% of populations have caries, with prevalence rates as high as 70% in some underserved, marginalised and vulnerable communities. However, the global silence on an epidemic affecting children worldwide has led to little dramatic actions taken to address it. A global caries prevention agenda for children is important to help set up the needed momentum for action. The absence of this global agenda makes prioritisation of oral disease in developing countries of Africa like Nigeria difficult as national actions are often led by global calls made by global organisations; the most viable being calls made by the World Health Organisation.

Looking forward into the next decade, the priority of the international dental community should be to support national efforts to understand their country’s caries epidemic. The outcomes of the finding would help countries like Nigeria plan for effective interventions at managing the caries epidemic in children should use the best possible combination of behavioural, biological and structural interventions approaches and recognise the importance of micro-, meso-, exo- and macrosystems on oral health. Efforts should essentially promote support for modification of the broader social factors that affect children’s oral health, including those that limit caries prevention activities and prompt detection of lesions [105].

Operational research should be conducted to test models of service provision for children. These models should ensure children’s prompt access to healthcare delivery services in a manner that encompasses all relevant systems, delivered through appropriate structures, and using appropriate technologies. A “one size fits all approach” would not be appropriate. Approaches would probably vary between States and Local governments. However, having a defined global agenda can help nations around the world focus on strategies that would help meet a global goal. Actors in the field of child dental health can then amplify this call as advocates.

## Summary

The national oral health policy in Nigeria recognises the need to increase awareness of all Nigerians for oral healthcare, to ensure equitable distribution and sustenance of essential oral health services at all times, to provide guidelines for standardisation of facilities, as well as programmes for continuing education for oral health personnel, and to promote the integration of oral healthcare delivery into all relevant health programmes, including primary healthcare. The policy therefore creates the enabling environment to advocate for design and implementation of a national oral health programme that combines approaches that are sensitive to the sociocultural context of the lives of individuals [106].

Addressing the oral health needs of the child is an ethical imperative. The current caries epidemic in the child is preventable, costly to society and cost effective to remedy. Caries is a disease that would benefit from the efficient use of existing societal resources in effective ways. Unfortunately, children are dependent and vulnerable to choices adults make for their lives. The current silence on this childhood epidemic needs to be broken. There is a strong need to call for action on caries.

## Competing interests

The authors declare that they have no competing interests.

## Authors’ contributions

MOF initiated the idea for the manuscript, wrote the initial framework, and edited the manuscript. NMC and NO assisted in the writing of the manuscript. OO and AAA supervised and edited the manuscript. All authors have read and approved the final manuscript.

## Pre-publication history

The pre-publication history for this paper can be accessed here:

http://www.biomedcentral.com/1472-6831/14/76/prepub
